# Trypsin induces an aversive response in zebrafish by PAR2 activation in keratinocytes

**DOI:** 10.1371/journal.pone.0257774

**Published:** 2021-10-08

**Authors:** Abdullah Alsrhani, Revathi Raman, Pudur Jagadeeswaran

**Affiliations:** Department of Biological Sciences, University of North Texas, Denton, TX, United States of America; North Carolina State College of Veterinary Medicine, UNITED STATES

## Abstract

Previously we have shown that trypsin, a protein typically involved in digestion, is released from gills of both fresh and saltwater fishes into surrounding water under stress or injury. We have also shown that each species produces trypsin with different specific activities. In this report, using zebrafish as a model, we identified that trypsin induces an aversive response in zebrafish larvae and adult zebrafish. Since Protease-Activated Receptor 2 (PAR2) responds to trypsin, we tested whether the aversive response is dependent on the activation of PAR2 located on the zebrafish skin cells. Zebrafish larvae treated separately with neomycin and zinc sulfate also showed aversive response indicating neuromast, and olfactory cells are not involved in this aversion. Cultured keratinocytes from zebrafish showed a response to trypsin. Zebrafish larvae subjected to knockdown of *par2a* also exhibited reduced escape response. Similarly, *par2a*-deficient mutant larvae displayed no response to trypsin. Since it has been shown that stress activates PAR2 and sends signals to the brain as shown by the increased *c-fos* expression, we tested *c-fos* expression in adult zebrafish brains after trypsin treatment of adults and found enhanced *c-fos* expression by qRT-PCR. Taken together, our results show that the trypsin activates PAR2 on keratinocytes signaling the brain, and this pathway of trypsin-induced escape response will provide a unique communication mechanism in zebrafish. Furthermore, since PAR2 activation also occurs in pain/pruritus sensing, this model might be useful in elucidating components of signaling pathways in pain/pruritus.

## Background

Trypsin is a digestive enzyme that is a serine protease and released as a zymogen from the pancreas into the duodenum and activated by enterokinase in the small intestines [1]. Previous studies have shown that trypsin activates PAR2 in human cells as well as in cells from other mammals generating a tethered ligand. In keratinocytes, the PAR2 stimulation by tethering peptides results in the release of TSLP, a cytokine that elicits an itch response [2, 3]. Likewise, PAR2 activation has also been shown to elicit neurogenic inflammation and is involved in pain-sensing [4–6]. Since PAR2 activation results in pain/itch sensing and trypsin activates PAR2, trypsin could cause itch or pain-like mechanisms.

In our previous report, we have shown that trypsin is released from gills under different stresses such as temperature, pH, overcrowding, and injury [7]. We have also shown all fishes produce trypsins as demonstrated by their ability to cleave a trypsin substrate [7]. Our initial hypothesis was that trypsin being an evolutionary precursor for coagulation proteases, would be involved in protecting gills from bleeding by inducing hemostasis. However, since trypsin release is not injury-specific and is released when subjected to a variety of different stresses, we hypothesized that there must be additional roles for the trypsin; otherwise, the organism would not waste the energy by producing trypsin to release it in the surrounding water.

Thus, our alternate hypothesis is that it may be communicating its stress to other fish via a trypsin signal. In this paper, we tested the hypothesis that fish may be and found that fish are aversive to trypsin and that this response is via a protease-activated receptor 2. This finding provides a novel mechanism in fish communication.

## Methods

### Fish husbandry

The zebrafish (*Danio rerio*) maintenance was according to the previously published protocols [8]. All procedures were approved by the Institutional Animal Care and Use Committee of the University of North Texas, and animal experiments were performed with humane care in compliance with the institutional guidelines.

### Trypsin chromogenic assay

The amount of trypsin was estimated by the chromogenic assay using the S-2238 substrate [7]. The assay was performed using 5 μl of trypsin in a final assay volume of 200 μl containing 500 μM S-2238 in 50 mM Tris-HCl pH 7.2. The reaction was incubated for 20 minutes, and the yellow color was measured at 405 nm in a 96-well kinetic microplate reader.

### Trypsin induction by trypsin

Individual wild-type adult zebrafish were kept in plastic cups containing 15 ml E3 medium (E3M), 5 mM NaCl, 0.17 mM KCl, 0.33 mM CaCl_2_, 0.33 mM MgSO_4_, pH 7.2. 195 μl of E3M was collected 20 seconds after placing the fish in this medium and used as baseline control. The fish was then removed and kept in 1 L of E3M to relieve stress. After 30 minutes, the same fish was placed in another cup of 15 ml of E3M containing 5 μl of 20 μM bovine trypsin. 195 μl of this trypsin containing E3M was collected 20 seconds before and after placing the fish. Trypsin activity was assayed in the E3M collected before and after trypsin treatment, and the activity in the sample collected before was subtracted from the sample collected after trypsin treatment which yielded trypsin activity induced by trypsin.

### Trypsin-mediated escape

Twenty μl of either 2 μM recombinant zebrafish trypsin or 20 μM bovine trypsin was added at the right end of a channel of a multiple channel tray containing 4.5 ml of E3M. Zebrafish larvae of age 7 dpf were kept in 0.5 ml of E3M and were transferred to the middle of a channel. Similarly, different control proteins, bovine serum albumin (BSA), Russell’s viper venom-Xa (RVV-Xa), and collagen were used. L-cysteine concentrations were according to the published methods. The larvae were photographed by a cell phone camera 30–60 seconds after the addition of the reagents. For quantification of the escape response, we counted the number of larvae that were on the left and right sides of the channel’s midpoint. At least 70% of the total larvae on the left side indicated a positive response. We used a 75 cm x 10 cm x 4 cm plexiglass tank filled with 2.5 L of E3M for the adult fish trypsin repelling experiment. At one of its ends, 2 ml of 10 μM recombinant zebrafish trypsin was placed, and approximately 6-month-old zebrafish were kept in the middle.

### *par2a* knockdown

We used a piggyback method for *par2a* knockdown. Zebrafish *par2a*-specific antisense oligonucleotide (aSO) and a control sense oligonucleotide (cSO) were designed using Primer 3 software. The oligonucleotides aSO (5′-GACTATATCCATGTTGGTGACCTTCTATA-AATTGTAACTG-3`), and cSO (5′-GAAGGTCACCAACATGGATATAGTCTATAAATTGT-AACTG-3′) were purchased from Invitrogen, Carlsbad, CA. Control vivo morpholino cVMO (5′-CCTCTTACCTCAGTTACAATTTATA-3′) was purchased from Gene-Tools LLC, Philomath, OR. The hybrid was prepared by mixing 4.5 μl of 0.5 mM cVMO with 4.5 μl of 0.5 mM of either aSO or cSO and 1 μl of 10X oligo-hybridization buffer containing 500 mM NaCl, 10 mM Tris–HCl (pH 8.0), and 1 mM EDTA (pH 8.0). Hybrids of aSO/cVMO and cSO/cVMO were prepared by heating the above mixture to 90°C and slowly cooling to room temperature using the Takara PCR Thermal Cycler.

5 dpf larvae were microinjected either with 4 nl aSO/cVMO or cSO/cVMO hybrids. Intravenous microinjection was made at the site where the inferior vena cava delivers blood to the yolk sac by using the Picospritzer III (Parker Precision Fluidics, Hollis, NH) together with a manual micromanipulator and an Olympus inverted microscope equipped with left- and right-hand Leitz micro-manipulators. After injection, the larvae were transferred to a petri dish containing system E3M, and after 48 hours, the larvae were subjected to the trypsin escape assay.

### Preparation of brain samples from fish after trypsin treatment

Individual adult zebrafish of similar size were kept for 30 minutes in separate plastic cups containing 15 ml of E3M. The fish were then treated with bovine trypsin (Sigma-Aldrich) or control BSA at a final concentration of 20 μM for 1 minute. The trypsin exposure times were staggered for each fish to enable a consistent exposure period from treatment to brain extraction. The whole-brain of adult zebrafish was obtained by dissecting under a dissection microscope as described previously with minor modifications [9]. All the micro-dissection tools were cleaned with 70% ethanol. Adult zebrafish were anesthetized by placing them in 0.15 mg/ml buffered tricaine methanesulfonate, pH 7.0 (Sigma-Aldrich, St. Louis, MO) until the fish lost equilibrium and tilted sideways (about 2 minutes). The fish was then placed on a flat plastic surface, the head of the fish was cut at the brain stem, the skin was removed, the skull was opened by using thin forceps, the brain was exposed, cranial nerves were cut, and the whole brain was gently removed to minimize the damage. The brain was then washed with ice-cold PBS (Phosphate buffered saline pH 7.0) for use in subsequent experiments.

### Skin cell cultures

The adult zebrafish were anesthetized as described above. The mucus layer was removed by first wiping with an alcohol swab and then using a scalpel blade to scrape off the scales gently. These scales were placed in a 50 ml tube containing 20 ml 1X PBS and washed three times by centrifuging at 5000 rpm in Sorvall Legend X1R centrifuge for 3 minutes. Subsequently, the scales were transferred to a tissue culture plate, allowing them to adhere to the culture dish for 2 minutes before gentle addition of the culture medium containing 90% L-15 medium (Sigma-Aldrich, St. Louis, MO), 10% FBS (Gibco, Carlsbad, CA) and 1.5 ml/100 ml Penicillin/Streptomycin (Gibco, Carlsbad, CA), pH 7.5 [10]. The plate was incubated overnight at 28°C, which allowed the cells to migrate from the scales attached to the culture dish. After 24 hours, the scales were gently removed with a pair of forceps, and the cells were lifted from the plates using cell culture scrapers. These cells were kept in 1X PBS or L-15 medium lacking phenol red for further experiments. For experiments involving microscopic observations, the cells were cultured on a Lab-Tek tissue culture chamber/slide (Miles Scientific, Naperville IL), were not scraped, and were used as described below.

### Calcium labeling and flow cytometry

For calcium labeling of cells, the cells were obtained as described above. They were then washed twice with sterile serum-free L-15 medium and collected in Eppendorf tubes. A mixture was prepared to consist of 1 μl of 1 mM Fluo-4 AM (Molecular Probes by Life Technologies, Eugene, OR) in DMSO with 1 μl of 20% Pluronic F-127 (Sigma-Aldrich, St. Louis, MO) and 1 ml sterile serum-free L-15 medium. 1 ml of this mixture was added to the cells and incubated in the dark at 28°C for 40 minutes. After incubation, the medium was aspirated, and the cells were washed with calcium-free 1X PBS (Gibco, Grand Island, NY) for use in further experiments.

The calcium-labeled cells were transferred to L-15 medium (without phenol red) and were placed in an Eppendorf tube in BD Accuri C6 flow cytometer (BD Biosciences, Franklin Lakes, NJ). The baseline Ca^2+^ level of the fluorescence-labeled cultured cells was measured for 1 minute. Subsequently, to the cells in the tube, 1 μl of 20 μM of bovine trypsin was added using a gel-loading pipette tip (STARLAB, Switzerland) without pausing the data acquisition. The calcium release was recorded over a few minutes. Fluo-4 AM signals were acquired in FL1-A. The raw data files were obtained using BD Accuri C6 Software (BD Biosciences, Franklin Lakes, NJ).

### RNA extraction

Total RNA was isolated using TRI Reagent (Sigma-Aldrich, St. Louis, MO)/1-bromo-3-chloropropane/isopropanol method from the above-cultured cells kept in 1X PBS. The suspension was centrifuged at 500 g for 5 minutes to pellet the cells. The pellet was then suspended in 400 μl of the TRI Reagent. The cells were homogenized using Polytron PT 10/35 Homogenizer with a controller (Kinematica, Switzerland) and centrifuged. The aqueous phase was precipitated using isopropyl alcohol. The dry pellet was then suspended in RNase-free water. Total RNA concentration and purity were determined using a NanoDrop. For isolating total RNA from the brain, TRI reagent was added according to the wet weight of the brain as recommended by the manufacturer. The brain tissue was then gently homogenized, and the homogenate was used to isolate RNA as described above.

### RT-PCR

The deoxyoligonucleotide primers for RT-PCR were purchased from Invitrogen (Carlsbad, CA). The primers used for amplifying *krtt1c19e*, *α-actin* positive control, and *par2a* receptor RNAs were as follows: forward krtt1c19e (5’-CTCTTGAGAAAGCCAATGCTG-3’), reverse krtt1c19e (5’-ACCTGTCCACTCA-TTTGATCG-3’), forward *α-actin* (5’-TGGCATTGCTGACCGTATGC-3’), reverse *α-actin* (5’-GTCATGGA-CGCCC-ATTGTGA-3’), forward *par2a* (5’-TCACCTGCCATGA-TGTCACT-3’), and reverse *par2a* (5’-TCTGAACGATGCTGGGATCA-3’). RT-PCR was performed on RNA using the SuperScript One-Step RT-PCR System with Platinum *Taq* DNA Polymerase (Invitrogen, Carlsbad, CA) and Takara thermal cycler. The RNA was converted to cDNA at 50°C for 30 minutes, followed by initial denaturation at 94°C for 1 minute. Following that, the amplification was carried out for 35 cycles of 30 seconds at 94°C, 1 minute at 55°C, 4 minutes at 72°C, followed by final extension of 7 minutes at 72°C and final cooling at 4°C. The resulting amplified DNA was resolved by 1.2% agarose gel electrophoresis along with size markers. The gel was stained with ethidium bromide and photographed. The intensities of DNA bands corresponding to *par2a* and *α-actin* were measured using ImageJ software, and the *par2a* band intensity was divided by the *α-actin* band intensity to yield a relative ratio of intensities.

For amplifying zebrafish *c-fos* transcripts, we used forward primer: (5′-AACTGTCACGGCGATC-TCTT-3′) and reverse primer: (5′-GCAGGCATGTATGGTTCAGA-3′). RT-PCR was performed, and the cDNA products were analyzed by agarose gels as described above. *ef1-α* cDNA was amplified as internal control with the forward primer (5′-CGGTGACAACATGCTGGAGG-3′) and reverse primer (5′-ACCAGTCTCCACACGACCCA-3′). The band intensities were measured using ImageJ software for comparing mRNA levels. All primers used in RT-PCR reactions were purchased from Invitrogen, Carlsbad, CA.

### Real-time RT-PCR analysis

*c-fos* mRNA was quantified by using a Bio-Rad iQ5 machine on mRNA from both target samples (trypsin-treated) and control (non-treated), which were prepared from the zebrafish brain as described above. The same set of *c-fos* primers described above was used along with SYBR Green dye in each reaction mixture. Fold change in *c-fos* transcripts determined by quantitation of cDNA from treated samples relative to an untreated (calibrator sample). The ef1-α cDNA was used as the endogenous control for the normalization of initial RNA levels. The relative gene expression was plotted using the calculation based on fold changes (ΔΔCt) [11].

### Diffusion of trypsin through mucus

The trypsin diffusion through mucus was performed as follows. In a well of 96-well plate and final assay volume of 400 μl, we placed 500 μM of S-2238 in 50 mM Tris-HCl pH 7.2 such that the well is full. A wet cheesecloth was folded into two layers and was cut to fit on the top of the well, so it was in contact with the substrate solution. Mucus was scraped from the lateral sides of the anesthetized fishes and was placed on the top of the cheesecloth to cover the entire area. 3 μl of 20 μM bovine trypsin was placed on the mucus layer. Immediately after placing the trypsin, the cheesecloth was removed, and the yellow color was quantified by measuring absorbance at a wavelength of 405 nm and comparing it to the absorbance of trypsin added to the substrate directly. In place of trypsin, BSA was used as a control.

### Statistical analysis

Statistical analysis was performed using the web-based program with the URL http://astatsa.com. Statistical significance was assessed by one-way ANOVA and by Holm-Sidak post-hoc analysis. A p-value < 0.05 was considered significant. The error bars represent the standard error of the mean.

## Results and discussion

**Fig 1:**
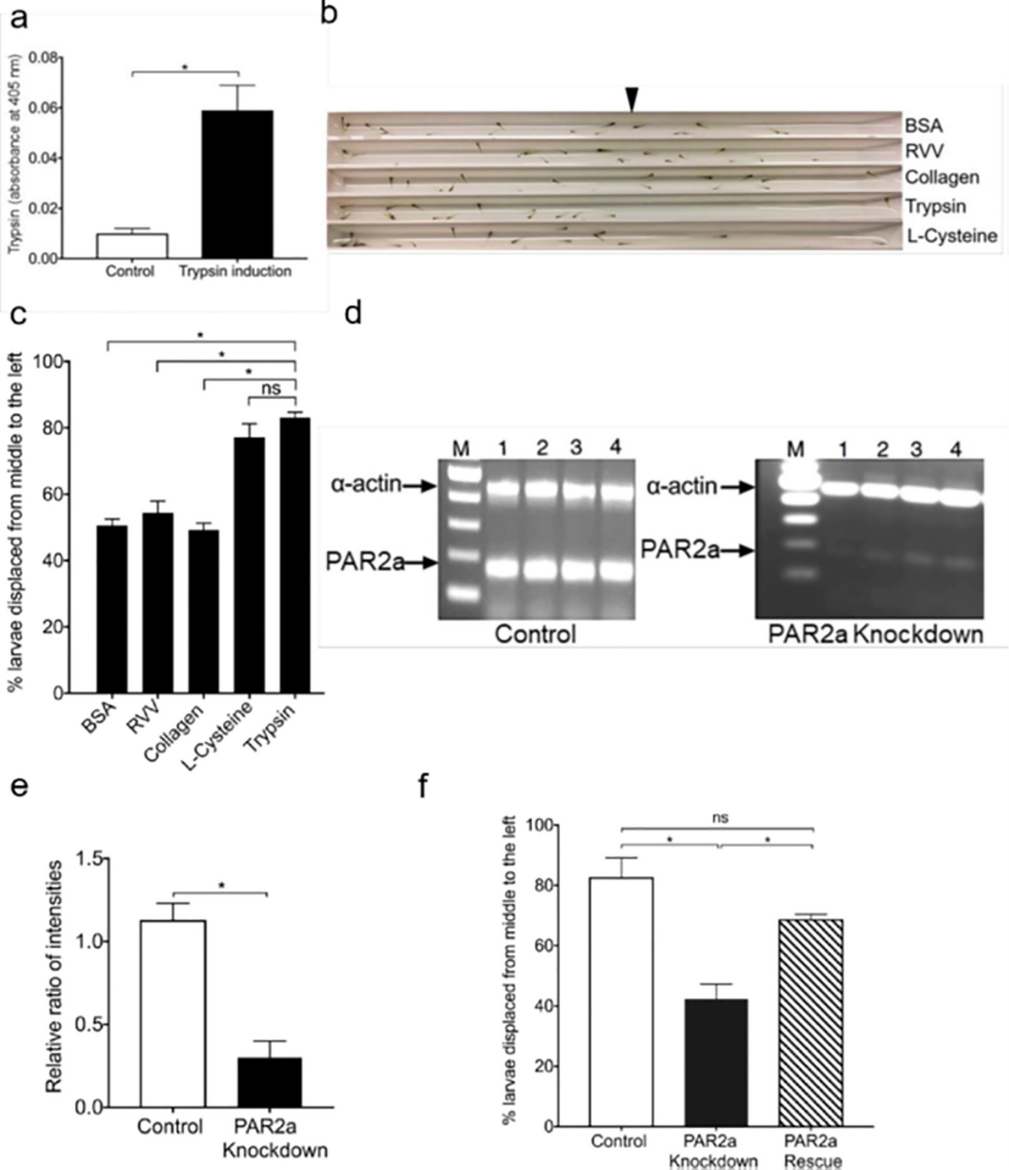
Aversive responses of zebrafish to trypsin. **a**. Trypsin released by zebrafish in response to trypsin itself. Absorbance at 405 nm represents the yellow color generated by digestion of the s-2238 substrate by trypsin. (n = 6 independent experiments; For each experiment 1 fish was used; *p<0.001). **b**. A representative photograph showing the dispersion of larvae in the elongated mini-tanks after the larvae were introduced to the tank at the midpoint (arrowhead) followed by injection of test chemicals on the rightmost end of each tank. The test chemicals were BSA, RVV, collagen, trypsin, and L-cysteine. Note the movement of larvae to the left side of the tanks away from trypsin and L-cysteine. **c.** Quantification of dispersion as the % larvae displaced from the middle to the left side in the mini-tanks in response to BSA, RVV, Collagen, Trypsin, and L-Cysteine. (n = 6 experiments; *p<0.001; ns, non-significant). **d.** *par2a* transcripts were decreased markedly in the *par2a* knockdown zebrafish larvae (7 dpf). Results of RT-PCR with *α-actin* serving as the internal control and DNA size markers in lane M. Lanes 1–4 each represent mRNA from one animal. Control and *par2a* knockdown embryos were injected with sense cSO/cVMO and antisense aSO/cVMO hybrids for the piggyback knockdown. **e.** Intensity ratios for *par2a* and *α-actin* bands. *par2a* band intensity was divided by *α-actin* band intensity and plotted on Y-axis as a Relative ratio of intensities. Note the *par2a* knockdown ratios are significantly less than that of control samples (n = 6 individual larva; *p<0.001). **f.** Rescue of trypsin repelling in *par2a* knockdown larvae. Y-axis shows % larvae displaced from the middle to left side of the tank after trypsin treatment of control, *par2a* knockdown, and *par2a* rescued larvae (n = 6 experiments; *p<0.001; ns, non-significant).

In our previous report, we have shown that trypsin is produced upon stressing the fish. To determine whether trypsin would induce the fish to produce more trypsin, we placed a single zebrafish in the E3M and exposed it to zebrafish trypsin. Then we assayed the resulting E3M for the release of new trypsin, which we predicted would be above background levels. As expected, we found the trypsin was more after induction by trypsin (Fig 1A). Since zebrafish released more trypsin into E3M upon exposure to trypsin, we hypothesized that the fish are responding to trypsin. We then devised a mini tank containing E3M, added recombinant zebrafish trypsin in the right end of the tank, and then placed zebrafish larvae in the middle of the tank immediately. Approximately 80% of larvae moved away from the midpoint in the mini tank (Fig 1B, S1 Video). Bovine trypsin had the same aversive effect except that it required ten times more concentration of bovine trypsin than the zebrafish trypsin. In contrast, when we used non-proteolytic proteins such as bovine serum albumin (BSA), collagen, and EcoRI, larvae distributed more or less equally on both sides of the midpoint of the tank. Similarly, when we used another proteolytic enzyme RVV-Xa (RVV), that cleaves coagulation factor X zymogen to generate active Xa, larvae also distributed approximately equally on either side of the midpoint in the mini tank. The trypsin-mediated fish aversion was similar to the positive control L-cysteine that has been shown to repel the fish. Fig 1B shows the aversive response of the larvae. This response was quantified as the ratio of the number of larvae on the left versus right half of the tank. The escape response was about 80% for trypsin and L-Cysteine and roughly 50% for control substances (Fig 1C). We obtained a similar aversive response in adult fish (see S2 Video), where they all moved away from trypsin (in this experiment, trypsin is placed on the left side) and clustered together. In the above experiments, the aversion response was usually within 30 seconds in both the larvae and adults but lasted in the larvae for 5 minutes, and in adults, it lasted for 3 minutes.

Since PAR2 is a substrate for trypsin, we tested whether trypsin elicits the aversive response via PAR2-mediated signaling [12, 13]. We performed knockdown of *par2a* by the piggyback knockdown method. RT-PCR results showed that *par2a* RNA was degraded by more than 75% (Fig 1D, S1 Fig and Fig 1E). When trypsin repelling was conducted, about 40% of the *par2a* knockdown larvae moved to the left side, in contrast to 80% obtained in normal larvae (Fig 1F). This percent repelling is approximately equal to the percentage of repelling in controls shown in Fig 1C. We also injected into embryos the RNA derived from the *par2a* cDNA driven by the T7 promoter and found results similar to the wild-type controls. These results suggest that the observed knockdown effect is specific to *par2a* (Fig 1F).

**Fig 2:**
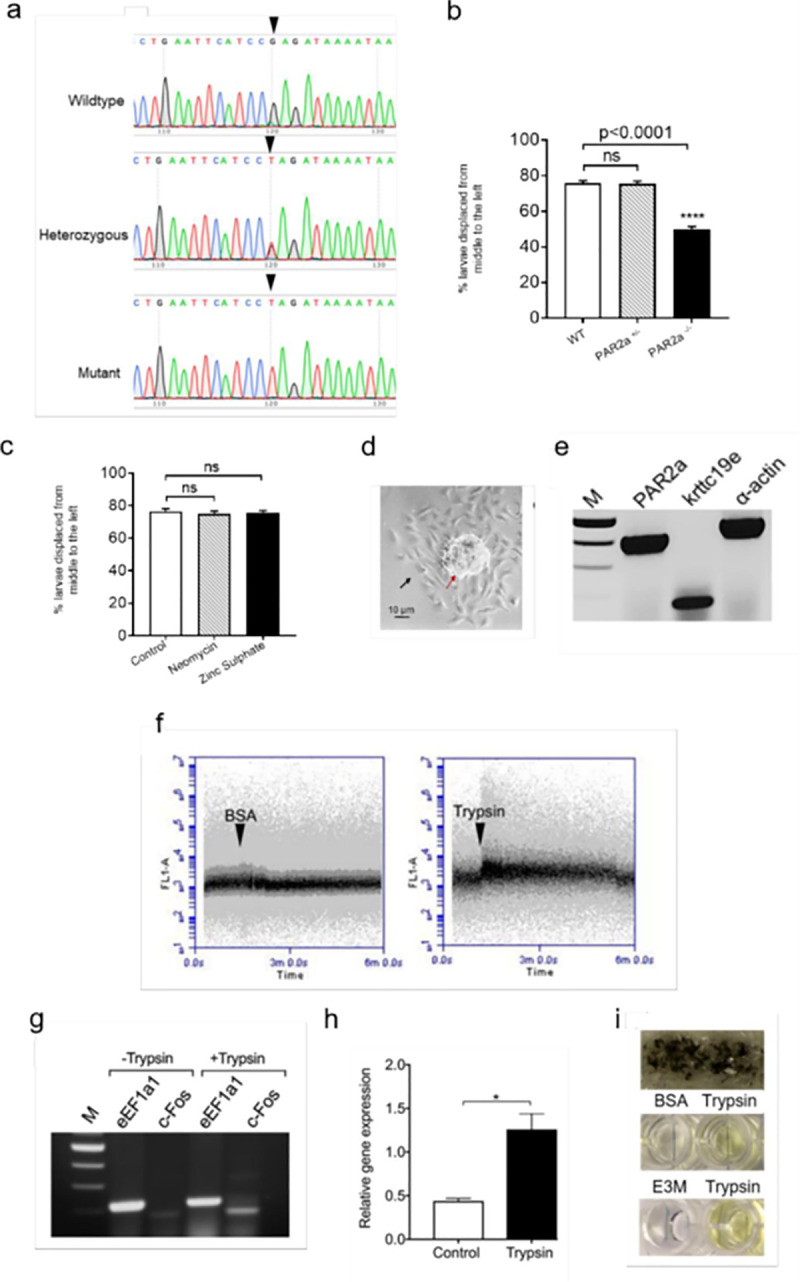
Knockout of *par2a* and detection of cell type and the components that sense trypsin signaling. **a.** DNA sequences of *par2a* gene of the wild-type (top), heterozygous (middle), and homozygous knockout mutants (bottom) are shown as chromatograms. The arrowhead marks the mutation. Only sequences around the *par2a* gene mutation are shown. **b.** The relation between trypsin repelling results and *par2a* knockout mutants. Showing for each genotype (WT, wild-type; *par2a+/ter*, heterozygote; *par2ater/ter*, homozygous knockout) the percent of larvae displaced from the middle to the left of the mini-tank opposite to trypsin administration. **c.** Evidence that the trypsin-sensitive surface cells are not neuromast or olfactory cells. Comparison of intact larvae and larvae with neuromast cells ablated by neomycin treatment and olfactory cells inactivated by zinc sulphate. Similar percentages of larvae from three groups were displaced from the middle to the left, away from the source of trypsin, suggesting that neuromast cells and olfactory cells were not responsible for the repellent effect of trypsin. (n = 6 experiments; ns, non-significant). **d**. Phase-contrast image of skin cell culture. Red and black arrows show the scale and skin cells, respectively. **e.** Confirmation of keratinocytes. RT-PCR products of three genes *par2a*, *krittc19e*, and *α-actin*. M, DNA size markers. Positive amplification of *krittc19e* and *par2a* showed that they are keratinocytes having Par2a receptors. **f.** Calcium signaling in keratinocytes induced by trypsin. The left and right panels are the fluorescence shown by flow cytometry when induced by BSA and trypsin, respectively. **g.** Induction of *c-fos* transcripts by trypsin exposure of adult zebrafish. Photograph showing the RT-PCR products of the zebrafish brain RNAs that were not exposed to trypsin (-Trypsin) and were exposed to trypsin (+Trypsin). Lanes show M, DNA size markers, ef1-α labeled as eEF1a1 (internal control, and *c-fos* labeled as c-Fos. **h.** Quantification of the induction of *c-fos* transcripts by trypsin by real-time RT-PCR. The relative gene expression of *c-fos* in the control and trypsin-treated whole brain is given on the y-axis. **i.** Diffusion of trypsin through the fish skin mucus. In the photograph, the top panel shows mucus layered on the cheesecloth covering the well of the 96-well plate. The middle panel shows the yellow color generated by the cleavage of S-2238 by trypsin diffusion (Trypsin) compared to control (BSA). The bottom panel shows negative (E3M) and positive (Trypsin) control reactions performed in the wells.

We also obtained *par2a* mutants from ZFIN, bred them to obtain mutant homozygotes, and confirmed them by DNA sequencing (Fig 2A). These mutants carry a premature termination codon (Fig 2A). We then subjected the control larvae and homozygote mutants to the trypsin repelling assay and found the control and *par2a* homozygote larvae did not have any differences in the distribution of larvae (50% of larvae on each side). The results are shown in Fig 2B. These results confirmed the knockdown results and are consistent with the lack of escape response due to *par2a* mutation and showed that the trypsin was acting on Par2a of the fish.

The finding of the involvement of *par2a* in trypsin response suggested that the cells on which trypsin is acting must be surface cells such as neuromast cells or skin epithelial cells. We treated the larvae with neomycin, a standard drug that inactivates neuromast cells. In the trypsin escape response, the neomycin-treated larvae moved away from the trypsin, similar to the untreated wild-type controls (Fig 2C), suggesting that neuromast cells might not be the direct target of trypsin. Similarly, olfactory cells were eliminated by treating the larvae with zinc sulphate. The results were similar to the neomycin experiment (Fig 2C). We then cultured the skin epithelial cells from adult zebrafish and verified their identity as keratinocytes by detecting keratinocyte-specific RNA (*krittc19e*) using RT-PCR (Fig 2D and 2E, S2 Fig). We then performed flow cytometry on the cultured skin cells loaded with calcium dye and found the fluorescence increases after treating these cells with trypsin (Fig 2F). These results suggest that trypsin signals via calcium release in keratinocytes.

To test whether trypsin signaling reaches the brain, we tested whether exposure of zebrafish to trypsin results in increased *c-fos* expression in the brain because it has been shown that stress induces c-fos expression in the brain [14–16]. The results showed that *c-fos* mRNA levels increased in the total brain by 3-fold after the trypsin treatment of the fish, based on RT-PCR (Fig 2G, S3 Fig) and real-time qPCR (Fig 2H). To test whether trypsin crosses the mucosal barrier, we tested whether trypsin diffuses through the mucous by layering fish mucus on a cheesecloth that touched a solution containing s-2238 substrate and then performing colorimetric trypsin assay. The results showed that trypsin readily passes through the mucus (Fig 2J).

The above results taken together indicate that trypsin via PAR2 signaling induces an escape response in fish. Furthermore, since trypsin-PAR2 is an enzyme-substrate interaction, there is an inherent species-specificity because of the varying degree of activities of different fish trypsins, as shown in our previous report [7]. These results suggest the feasibility of fish communication based on trypsin release. For example, the larvae may be aversive to the trypsin produced by the adult fish so they can escape when an adult fish reaches to consume them. Interestingly the fish clustered closer together without being repelled away from the trypsin produced by the fish in the cluster. This clustering is possible because PAR2, a GPCR, is known to be desensitized due to continued ligand exposure and due to such desensitization since more trypsin is produced around the cluster because of more fish and so they will be able to cluster [17]. It is difficult to predict how much trypsin is produced in a school in a natural setting. We have previously estimated that a stressed fish produces 0.13 ng of trypsin in 10 seconds [7]. Thus, in a large school of fish, once the initial fish starts releasing trypsin, it probably communicates with the adjacent fish, so this neighboring fish produces trypsin. Such communication may result in a chain reaction producing more trypsin by all the fish in the school.

In conclusion, we report here a novel finding that fish are aversive to trypsin that is produced during stress. The trypsin that is released communicates with neighboring fish via PAR2 activation on the surface skin keratinocytes. Since PAR2-mediated signaling occurs in the pain/itch pathway, we could use this trypsin repelling model to understand the components of the pain/itch pathway.

## Supporting information

S1 Fig Raw image of Fig 1D. Raw images of agarose gels that are shown in Fig 1D are included.
(PDF)

S2 Fig Raw image of Fig 2E. Raw image of agarose gel that is shown in Fig 2E is included.
(PDF)

S3 Fig Raw image of Fig 2G. Raw image of agarose gel that is shown in Fig 2G is included.
(PDF)

S1 Video Aversive response of zebrafish larvae to trypsin. Video showing the larvae moving away from trypsin placed on the right side of the tank.
(MP4)

S2 Video Aversive response of adult zebrafish to trypsin. Video showing the adults moving away from trypsin placed on the left side of the tank.
(MP4)
